# The stability of conditional cooperation: beliefs alone cannot explain the decline of cooperation in social dilemmas

**DOI:** 10.1038/s41598-020-70681-z

**Published:** 2020-08-12

**Authors:** Luciano Andreozzi, Matteo Ploner, Ali Seyhun Saral

**Affiliations:** 1 Department of Economics, University of Trento, 38122 Trento, Italy; 2 Max Planck Institute for Research on Collective Goods, 53113 Bonn, Germany

**Keywords:** Human behaviour, Social evolution

## Abstract

An often-replicated result in the experimental literature on social dilemmas is that a large share of subjects choose conditionally cooperative strategies. Cooperation generated by such choices is notoriously unstable, as individuals reduce their contributions to the public good in reaction to other subjects’ free-riding. This has led to the widely-held conclusion that cooperation observed in experiments (and its decline) is mostly driven by imperfect reciprocity. In this study, we explore the possibility that the type of reciprocally cooperative choices observed in experiments may themselves evolve over time. We do so by observing the evolution of subjects’ choices in an anonymously repeated social dilemma. Our results show that a significant fraction of reciprocally cooperative subjects become unconditional defectors in the course of the experiment, while the reverse is rarely observed.

## Introduction

The large experimental literature on social dilemmas, such as the Public Goods Game (PGG), the Prisoner’s Dilemma (PD) and the Trust Game (TG), has been remarkably coherent in revealing two stylized facts. First, subjects cooperate more than they would do if they were rational and purely egoistic. Second, when the game is repeated anonymously, cooperation declines over time, although it rarely disappears altogether. The early explanations for this phenomenon can be grouped under two headings: the *learning hypothesis*^1,2^ and the *conditional cooperation hypothesis*^3,4^.

According to the learning hypothesis, subjects are mostly self-interested, but they are boundedly rational and it takes time for them to learn the workings of the game they are playing. Proponents of this approach believe that what is observed in the early stages of any experiment involving a social dilemma is a mixture of noise and confusion^1,5^. An obvious alternative to the learning hypothesis was that the cooperation observed in the lab and its decline could be the consequence of some form of other-regarding preferences like reciprocal fairness^6–8^ or inequity aversion^9,10^.

Social-preference models introduce an important difference in the analysis of social dilemmas. From the point of view of a rational and selfish player, the PGG and the PD are extremely simple games to play: zero contribution to the public good is the optimal strategy, regardless of the choice made by other players. An individual who is partially motivated by inequity aversion or reciprocal fairness, instead, sees social dilemmas more like coordination games: She is willing to cooperate, provided that other subjects cooperate as well. Notice that since for an individual of this type the optimal choice depends upon her beliefs about other players’ behavior, learning is going to play a role. When a game is played more than once, reciprocally motivated subjects learn about the social behavior of others, for example whether they are selfish or cooperative, and adapt their choices accordingly. To distinguish these two types of learning, we shall refer to the one invoked by the learning hypothesis as *adaptive* learning^11^, and the one invoked by the reciprocity hypothesis as *social* learning^12^.

In two seminal papers, Fischbacher et al.^3^ (FGF) and Fischbacher and Gächter^4^ (FG) pioneered the use of the strategy method to test the implications of social-preference models in the PGG. They elicited subjects’ contributions to the public good, conditional on the contribution by the other players in their group. Their data revealed that around half of the subjects could be classified as conditional cooperators: They were willing to contribute to the public good only if other subjects were contributing as well. Unconditional defectors comprised about 30% of the total. These findings revealed that the cooperation generated by social preferences is inherently fragile because most subjects display a “self-serving bias”: They are almost never willing to give more, and frequently give less, than what they expect others to give. It follows that cooperation would unravel over time, even in the absence of selfish free-riders, either because of this self-serving bias or because of a coordination failure due to heterogeneous prior beliefs^13^.

The conditional cooperation hypothesis was immediately recognized as a particularly strong candidate to explain the experimental evidence on the PGG, as it provided a unified explanation both for the initially high levels of cooperation and for its subsequent decline. Furthermore, these experimental findings proved to be robust^14–21^ and this contributed to making it the dominant explanation in the experimental literature.

The explanation of the decline of cooperation based on reciprocity hinges on the hypothesis that the social preferences revealed with the strategy method remain stable for the entire duration of the experiment. What does change during the experiment are subjects’ beliefs about the cooperative choices of their opponents.

FGF were well aware of this issue. In order to check the stability of preferences, they conducted a post-experimental test with a hypothetical contribution table. They noticed that the results from the hypothetical choices were almost identical to subjects’ actual choices in the experiment. The follow-up paper, FG, contains a much more thorough discussion of this issue. The authors elicited conditional preferences either before or after a ten-period repetition of the standard PGG. Moreover, they elicited beliefs in order to predict subjects’ contributions from their conditional preferences. Their results showed, first, that the preference distributions of subjects’ conditional types were similar, whether the elicitation was done before or after the PGG. Second, they showed that the predictions from the beliefs and the conditional preferences were rather successful at explaining subjects’ choices.

As the question was seemingly settled, the literature that followed paid little attention to the stability of preferences. This was probably due to the fact that the few studies that explicitly tackled this issue appeared to be consistent in finding that preferences were stable over time^18,20^. Muller et al. for example, aim at disentangling the effect of learning and strategic behavior in PGG^18^. To this end, they consider a simplified version of the PGG, played for five periods with stranger protocol. Eliciting the preferences in each period, the authors find that “the distribution of the types is fairly stable”, although they also notice that almost two-thirds of their participants were classified differently in some periods in terms of conditional types. Volk et al.^20^ focus on the stability of conditionally cooperative preferences in a longer time frame. They elicit subjects’ preferences in three waves: first, two-and-a-half months after the first session, and then five months after the first session. Their data reveal that conditional preferences are remarkably stable. However, a closer look shows that the within-individual stability is not as solid: nearly 50% of all subjects were classified differently at least once in the three waves.

A proponent of the learning hypothesis may remain skeptical. A possible objection to the existing evidence is that too little effort has been made towards seeing to which extent reciprocally cooperative preferences are resistant to learning. Note, for example, that in the experiment run by FG each subject fills the conditional cooperation schedule only once, either before or after the experiment. This can hardly be considered an environment in which learning can take place.

Another reason for skepticism comes from more recent evidence, which shows that at least a share of the reciprocity observed in the lab may be due to an imperfect understanding of the game. Experiments reveal that subjects may condition their contributions on seemingly arbitrary elements, like the contributions made by other groups of subjects in unrelated experiments or even random numbers^22,23^, that a fraction of the subjects involved in social dilemmas display similar pro-social behavior in interactions with computers and human beings alike^24–26^, and that conditionally cooperative subjects are precisely those who seem unable to distinguish computers from human beings^26^. Since part of the reciprocally cooperative choices elicited with the strategy method seem to be due to the confusion of subjects, one should expect it to clear up as they become more familiar with the game they are playing. The decay of cooperation would then be explained, at least in part, not by belief updating, but by adaptive learning.

In this paper, we address these issues with an experimental design that aims at observing the evolution of the choices over conditionally cooperative strategies as the game unfolds. To do this, we run a simple social dilemma experiment in which conditionally cooperative strategies are elicited in each period of the game. In particular, in our design, subjects are asked to fill the conditional cooperation schedule not just at the beginning or at the end of the experiment, but after every period. This allows us to observe how subjects’ choices over reciprocally cooperative strategies evolve over time. Our results show that conditional strategies might not be as stable as they were initially thought to be, and those subjects who choose conditionally cooperative strategies are less stable in their choices than others. We observe a large fraction of subjects who are initially classified as reciprocal cooperators to switch to unconditional defection, while the opposite transition is virtually never observed. We postpone the discussion of these findings to the concluding section.

## Methods

**Figure 1:**
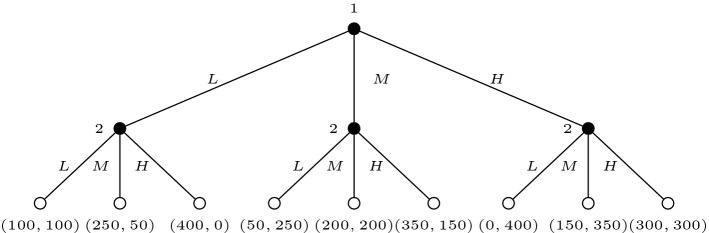
The three-actions sequential prisoner’s dilemma (3SPD) game.

We use a three-action version of a sequential Prisoner’s Dilemma, which is a two-player variant of the Public Goods Game used by FG and FGF. Each player receives 100 tokens and is given the opportunity to transfer nothing (low transfer, *L*), 50 tokens (medium transfer, *M*), or 100 tokens (high transfer, *H*) to the other player. The second player chooses after having observed the first player’s choice. A player’s final payoff is the sum of the tokens she did not transfer and the tokens she received by the other player, multiplied by three. The Pareto-optimal choice is thus to transfer 100 tokens, although the dominant choice is to transfer nothing. The extensive form of the game is represented in Fig. 1. We will refer to this game as the Three-Actions Sequential Prisoner’s Dilemma (3SPD).

We use the strategy method to elicit subjects’ choices over conditionally cooperative strategies as first and second movers. Before playing the game, each subject has to state which level of cooperation she would choose if she was selected as first mover. Also, she must choose a level of cooperation in response to any of the three levels of cooperation the first mover may choose, to be used if she is selected as second mover. After decisions are made, roles are randomly assigned and payoffs are obtained. To minimize the effect of repeated games, we use the stranger matching procedure: in each period, each subject is matched randomly and anonymously to another subject.

To test the extent to which subjects may respond to the strategies employed by the other subjects, we manipulate the information they receive about the conditional strategies of the others. In the baseline treatment, which we call *NoCondInfo*, subjects are given no information about the other subjects’ conditional choices. In the treatment we call *CondInfo*, each subject who was chosen as first player is also informed about the way in which the subject with whom she was matched filled the conditional cooperation schedule.

### Experimental design

In all treatments, we used the strategy method to elicit subjects’ strategies as both first and second movers. Subjects are informed that they will be selected in either role with equal probability. When choosing their strategy as the second mover, each subject has to select his preferred strategy, conditional upon the strategy chosen by the first mover. The screenshots of the interface can be found in *Supplementary Information*.

Subjects played the 3SPD game for 10 periods. In each period, subjects were informed that they would be randomly matched to play a game, and they disclosed their strategies both as first and as second movers. Before choices were made, we elicited each subject’s beliefs about their counterpart’s choice. In order to preserve incentive compatibility, we awarded correct guesses by using a quadratic scoring rule^27,28^. Additional information on belief elicitation can be found in *Supplementary Information*.

Before the experiment, subjects were required to answer questions aimed at ascertaining their comprehension of the 3SPD game. Subjects could not proceed to the next step if they failed to provide correct answers to all questions. After completing this stage, four training periods took place, in which subjects played against randomly responding computer players and practiced the belief elicitation procedure. After the training periods, the actual experiment took place: the subjects played ten periods of the 3SPD game against each other. Before the end of the experiment, we gave participants a short questionnaire that contained demographic questions.

### Treatments

In order to evaluate the effect of the information on the conditional strategies of the others, we presented subjects with the following information scheme in a between-subjects fashion: in one treatment (*NoCondInfo*), subjects were informed after each period whether they were the first or the second player, the action they played in the assigned role, the action chosen by their counterpart, and their payoff. In the second treatment (*CondInfo*), we also informed subjects whose roles had been determined as first movers about the conditional strategy chosen by their counterpart, who played as the second mover. Every other step is identical for the two treatments.

### Participants and sessions

The experimental sessions were conducted at CEEL, University of Trento. In total, 134 subjects participated in six experimental sessions (*NoCondInfo*: 3 sessions, 68 subject in total; *CondInfo*: 3 sessions, 66 subjects in total). A copy of the instructions that were handed out in printed form is available in *Supplementary Information*. All subjects were able to answer the control questions correctly. No subject or session was excluded from the data. Experiments were programmed and conducted in z-Tree^29^. The game consisted of ten periods and one period was randomly selected for payment. Participants were given 3 EUR each as a fixed payment and earned between 0 to 15 EUR in addition to that amount, according to the selected period in the session and their payoff in that period.

**Figure 2:**
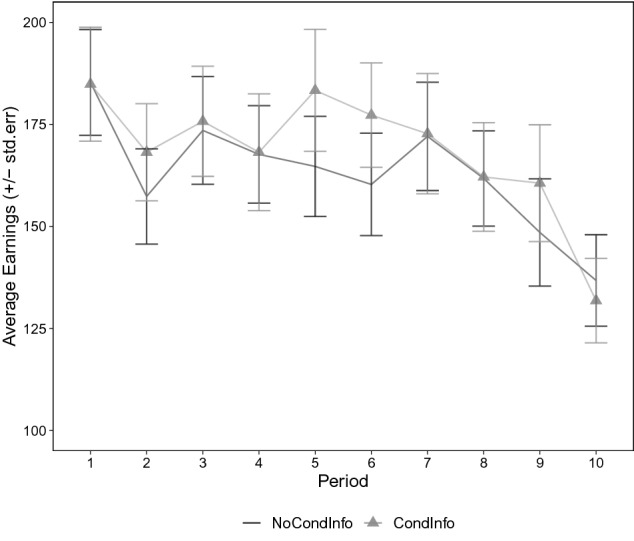
Average earnings over the periods show the decline of cooperation. Bars represent standard errors.

### Ethical approval

The procedures of the experiment are in accordance with the Helsinki Declaration of the World Medical Association and General Data Protection Regulation (GDPR) of the European Union. The experiment took place in a stable computer lab with a permanent subject pool. A written informed consent form was obtained from all participants. Given that in the present study participants were drawn from a regular subject pool (university students), the University of Trento exempted this study from IRB approval.

## Results

We focus on the conditional choices made by our subjects. Notice that, since all choices are made before roles are assigned, the choices made by all subjects are relevant, regardless of whether they were selected to play as first or second mover. We represent a strategy for the second player as a triple ABC, where A is the action chosen in response to L, B is the action chosen in response to M, and C is the action chosen in response to H. For example, LMH is the perfect conditionally cooperative strategy that always matches the first mover’s choice. Given our simple strategic setting, the classification of subjects is immediate. We distinguish *selfish* individuals (LLL) from *perfect conditional cooperators* (LMH) and *imperfect conditional cooperators* (LLM, LLH, LHH, MMH, MHH), that is, all the subjects other than perfect conditional cooperators, whose contribution is a monotonic function of the first-mover contribution. In our sample, there is a small group of *hump-shaped* contributors, whose contribution is higher in response to intermediate contributions by the other subjects (MHM, LHM, LML). All other subjects are labeled as “other patterns”. For more information about the classification, see *Supplementary Information*.

### First-period choices and the evolution of cooperation

Our first results are in line with the previous experiments on repeated social dilemmas: The majority of subjects can be classified as conditional cooperators and cooperation declines over time. In our sample, more than half of the subjects (67%) can initially be classified as conditional cooperators, while only a minority is choose unconditional defection (21.6%). Figure 2 shows the average payoff subjects receive in each period, in both treatments. As in most other experiments with similar games, payoffs decline over time as subjects switch to less cooperative strategies.

### The distribution of conditionally cooperative strategies changes over time

**Figure 3:**
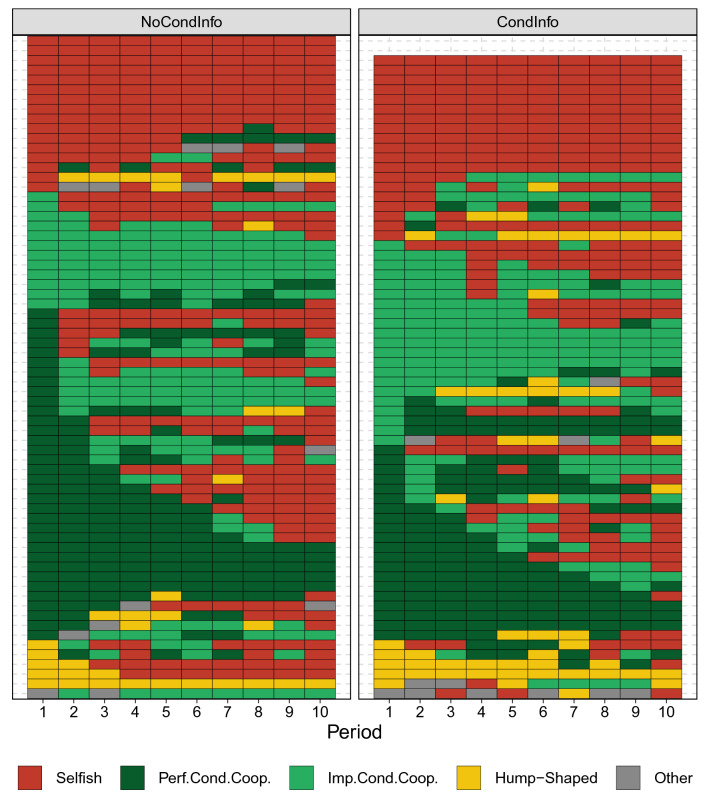
Subjects’ conditional strategies over ten periods for each treatment. Every row represents a subject and every colored rectangle captures his conditional strategy in the period on the x axis.

Figure 3 represents the evolution over time of the choices made by each subject as second mover, in both treatments. Each row represents a single subject over 10 periods. The red and dark green rectangles represent choices of subjects who are selfish (LLL) and perfect reciprocators (LMH), respectively. The light green rectangles pool together all imperfectly cooperative choices, while the yellow rectangles correspond to hump-shaped choices. All other patterns are represented by gray rectangles. This picture reveals that only a small minority of subjects keep the same strategy throughout the game (26%). Some of them repeatedly switch between several strategies, while others switch only once.

**Figure 4:**
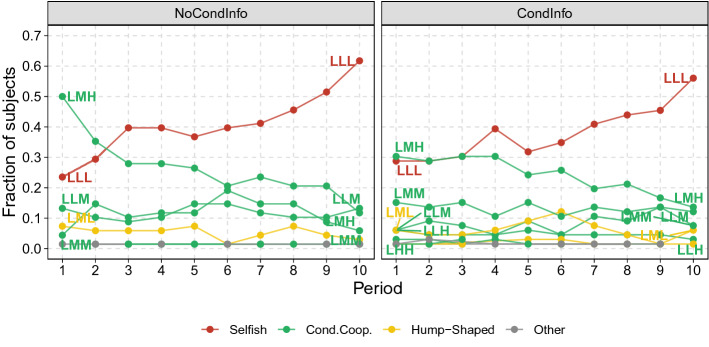
Fraction of subjects choosing each distinct strategy in each treatment.

Figure 4 shows the evolution over time of the number of strategies played by second movers in the two treatments. It reveals that the change in the composition of the population is mostly due to the decline of the number of subjects who choose the perfect reciprocating strategy LMH and to a corresponding increase in the number of subjects who choose unconditional defection (LLL). The frequency of all other strategies remains fairly constant across the periods.

### Perfect conditional cooperators are more prone to change over time

To investigate the stability of observed choices at individual level, we retain the customary classification based on the choices subjects make in the first period of the experiment. In this setting, to say that a subject is, for example, a perfect conditional cooperator only means that this is the choice she makes in the first period of a social dilemma experiment. We then define a measure of the *stability* of the chosen strategy. We say that the strategy type chosen in the first period is stable if it is also his modal choice in the last five periods. Otherwise, a subject is labeled as unstable.

**Table 1:** Stability according to the last five periods for each type and proportion comparisons.

	Num. of observations			Pearson’s chi-squared test p-value			
	Stable	Unstable	Percentage	Selfish	Perf. cond.	Imp. cond.	Hump-shp.
Selfish	27	8	77.1				
Perf. cond.	16	38	29.6	< 0.001			
Imp. cond.	16	17	48.5	0.028	0.123		
Hump-shp.	3	7	30	0.016	1.000	0.504	

Table 1 shows the fractions of stable subjects for different types according to this measure. The most stable subjects are those who turn out to be *unconditional defectors* (choosing LLL) in the first period. More than 3/4 of subjects falling into this category display stable choices. The least stable subjects are those who are initially *perfect conditional cooperators* (LMH). Less than 1/3 of subjects in this category are classified as stable. A series of chi-squared tests show that both *perfect conditional cooperators* and *imperfect conditional cooperators* differ from *selfish* subjects (p-value < 0.001 and p-value = 0.028, respectively). A significant difference in stability relative to the selfish type is detected for the *hump-shaped* (p-value = 0.016) subjects as well. All other comparisons do not detect any significant difference (all p-values > 0.123).

### Information has a limited effect on conditional strategies

A comparison between the treatments *NoCondInfo* and *CondInfo* reveals that it makes no difference whether subjects are shown the conditional choices of the other subjects. Table 2 shows the results of mixed effects logistic regressions where the dependent variable is a dummy variable capturing conditionally cooperative strategies. The results of the analysis indicate that the likelihood of using such a strategy decreases sharply over time (*Period*). However, the additional information on the counterpart’s conditional strategy does not have a significant effect on the choice of playing a conditionally cooperative strategy, as shown by the coefficients of *Treatment CondInfo* and *Period:Treatment CondInfo*. We performed a series of robustness checks on the regression outcomes of Table 2. In particular, we explored alternative model specifications to control for repeated observations at the individual and session levels. Results are consistent with those in Table 2 when estimating the same models, with random effects only at the individual level and only at the session level. Results are also confirmed by a generalized linear model with a two-way cluster-robust standard errors specification. Furthermore, a dynamic panel data estimate, taking into account the persistence of type, provides support to the negative effect of Period reported in Table 2. See *Supplementary Information* for robustness checks.

**Table 2:** Results of mixed-effects logistic regression models investigating the change in conditional cooperation and treatment effects.

	Model 1	Model 2
(Intercept)	1.48 (0.45)**	1.34 (0.48)**
Period	− 0.24 (0.03)***	− 0.22 (0.04)***
Treatment *CondInfo*	− 0.06 (0.59)	0.22 (0.67)
Period:treatment *CondInfo*		− 0.05 (0.06)
AIC	1225.76	1227.04
BIC	1251.76	1258.24
Log Likelihood	− 607.88	− 607.52
Num. obs.	1340	1340
Num. groups: subject	134	134
Num. groups: session	6	6
Var: subject (intercept)	9.79	9.82
Var: session (intercept)	0.02	0.02

Dependent variable is a binary variable indicating whether the individual uses a conditionally cooperative strategy (perfect and imperfect) or not.

***p < 0.001, **p < 0.01, *p < 0.05.

### Beliefs play a role in the evolution of conditional strategie

In order to understand the effect of beliefs on conditional strategy choices, we run separate mixed-effect logistic regression models for each type: in each model, the dependent variable is a dummy variable of the subjects’ strategy classification. These dummy varibles can be interpreted as the probability of a subject choosing a strategy of a certain type. Table 3 shows the probability of being classified as conditional cooperator, selfish and hump-shaped in different models. The beliefs on each choice made by the opponent are calculated as the expected transfer amount by the opponent for the specific choice. For instance, if the subject believes that the opponent will transfer 0 tokens with an 80% probability and 50 and 100 tokens with an equal probability of 10%, the expected transfer amount is 15 tokens. Then it is normalized to 0.15 according to the maximum expected transfer of 100 tokens. See *Supplementary Information* for details.

As Table 3 points out, subjects who expect others to respond to high transfer with a greater amount (response to H) are more likely to be conditional cooperators and less likely to be selfish. On average, an expectation of 10 tokens more by the opponent as a response to action H, increases the likelihood of being a conditional cooperator by around 40%. The subjects who believe that the other subjects start with a higher level of cooperation as a first move are more likely to be conditional cooperators as well. Our data also suggest that the subjects who expect a higher response to the action M are more likely to be hump-shaped contributors, however this result is only marginally significant (p-value = 0.049).

A comparison of the Akaike Information Criterion (AIC) and the Bayesian Information Criterion (BIC) in Tables 2 and 3 shows that incorporating beliefs on the specification improves the quality of the model, although it does not reduce the period effect substantially: In each period, on average subjects become 17% less likely to play a conditionally cooperative strategy and 20% more likely to play the selfish strategy. The results can be taken as evidence that neither the beliefs nor adaptive learning and selfishness alone can explain all the data: Switches among different types of conditional cooperation are partially due to subjects’ beliefs about other subjects’ choices.

**Table 3:** Results of mixed-effects logistic regression models investigating conditional types.

	Dependent variable		
	*isCondCoop*	*isSelfish*	*isHumpShaped*
(Intercept)	− 1.63* (0.76)	1.67* (0.78)	− 7.90*** (1.44)
Period	− 0.17*** (0.03)	0.20*** (0.04)	− 0.08 (0.06)
Treatment *CondInfo*	0.00 (0.58)	− 0.08 (0.64)	0.40 (0.94)
Belief—first player (Uncond.)	1.93** (0.71)	− 1.23 (0.71)	− 1.23 (1.35)
Belief—response to L	− 1.23 (1.25)	− 0.70 (1.27)	− 0.65 (1.95)
Belief—response to M	1.08 (1.14)	− 2.28 (1.18)	3.50* (1.78)
Belief—response to H	4.01*** (0.76)	− 3.73*** (0.79)	− 1.14 (1.19)
AIC	1120.56	1066.17	427.74
BIC	1167.36	1112.97	474.55
Log Likelihood	− 551.28	− 524.09	− 204.87
Num. obs.	1340	1340	1340
Num. groups: subject	134	134	134
Num. groups: session	6	6	6
Var: subject (intercept)	8.66	8.32	34.76
Var: session (intercept)	0.01	0.14	0.00

Dependent Variable in each column is a binary variable indicating whether the individual’s strategy is classified as the corresponding conditional type or not. Beliefs are measured in terms of expected transfers by the opponent for each distinct choice. Each belief measure is normalized to the interval [0,1].

***p < 0.001, **p < 0.01, *p < 0.05.

## Discussion

Our results are partly in contrast to those of Fischbacher and Gächter^4^. While in their experiment subjects are remarkably stable in choosing their conditional strategies, in ours a steady decline of reciprocally cooperative strategies is observed. At this stage, we can only speculate about the reason for this discrepancy, and we believe that more research is needed to settle this issue. We notice, however, that the most remarkable difference between the two experiments is in the number of times the choices over conditionally cooperative strategies are observed. While in the original experiment subjects fill out the conditional cooperation schedule only once, either before or after the ten periods of the game, in our experiment they fill it out ten times. Furthermore, and most importantly, these opportunities to change one’s own choices occur in each period, after the outcome of the stage game is revealed and payoffs are obtained. This probably induces subjects to consider their choices more carefully, gives them a better feeling for their consequences, and allows them to form more accurate beliefs about what other subjects are doing.

We conclude by putting forward what we believe are the three most promising explanations of why all these factors may produce the kind of choice instability we observe. A defender of the reciprocity hypothesis may argue that cooperators are inclined to reciprocate not only the *actions* chosen by their partners when playing as first movers, but also the type of conditionally cooperative preferences they reveal when playing as second movers. This is a subtle but crucial difference. Upon learning that the first mover has defected, a reciprocator playing as second mover will defect. This is the familiar consequence of reciprocity, which is captured by the strategy method and is deemed to be responsible for the decay of cooperation. On the other hand, upon learning that some of the other subjects are unconditional defectors (i.e., they would respond with defection to cooperation), a reciprocator may be induced to switch to unconditional defection as well. We shall call these two types of reaction level-one and level-two reciprocity, respectively. Clearly, the decay in the number of conditional cooperators may be due to level-two reciprocity, and should then be attributed to social, rather than adaptive, learning. This interpretation would corroborate the findings of those experiments^12,15^ that try to eliminate social learning by sorting individuals into homogeneous groups. A common finding in those experiments is that, when non-selfish players are allowed to interact among themselves (and hence social learning plays a smaller role), the cooperation rate remains high throughout the experiment.

A second explanation is that choice instability is mostly due to a combination of selfishness and adaptive learning. When the interaction is anonymous, unconditional defection is the optimal strategy, as a reciprocally cooperative subject fails to exploit the other subjects who play cooperatively as first movers. Selfish subjects who learn from experience will soon discover that when the setting is anonymous, there is no point in reciprocating the cooperative behavior of the first mover, and they will eventually become unconditional defectors.

Finally, part of our experimental results could be explained by repeated game effects, which may play a role even in the anonymous setting we consider (See, for example, Andreoni and Miller^30^ and Andreoni and Croson^31^. See Chaudhuri^32^ for a review. In our experiment, we followed FGF in using a stranger matching setting just because it minimizes repeated games effects.) Since the game is repeated 10 times among randomly matched subjects in the lab, in every period each subject has a non-negligible chance of meeting either an opponent she met in a previous period, or a subject who has interacted with one of her previous opponents. This may provide an incentive to subjects to be reciprocally cooperative in early periods, and switch to unconditional defection as the end approaches. According to this view, the decay in the reciprocally cooperative strategies would reflect neither a higher level of reciprocity, nor a process of adaptive learning. It would rather be a rational response to the kind of setting in which the interaction takes place. A legitimate question is to what extent, in the light of these considerations, what is observed in our experiment (where choices over strategies are made at every round, and hence repeated game effects are bound to play a larger role) is comparable with the results of the experiment run by FGF, in which choices are made only once, and hence these effects are less prominent. In the future, more refined experimental protocols will give us a better picture of the way in which reputations effects interact with adaptive learning to produce the kind of strategy evolution we observe in the data.

Naturally, our results may be driven by a combination of all these factors and, as we pointed out above, the evolution of beliefs we observe suggests that this is likely to be the case. However, the fact that the patterns in the *CondInfo* and the *NoCondInfo* treatments are similar suggests that adaptive learning plays a larger role than level-two reciprocity. To see why this may be the case, notice that in the *NoCondInfo* treatment a subject must *guess* what kind of conditional strategy the other subjects are choosing. For example, if she cooperates when in the first-mover position, she may discover that there are subjects who reply with defection to cooperation. If she always defects she will never discover that. By contrast, in the *CondInfo* treatment, there is no need to guess, as the information about the conditional strategy chosen by the opponent is provided by the experimenter. If this piece of information were determinant in the decision to switch to unconditional defection, one would expect the decline of reciprocity to be sharper in the *CondInfo* than in the *NoCondInfo* treatment. The fact that both treatments are indistinguishable reveals that information about the conditional choice made by the other subjects plays a minor role, if any.

Independently of which explanation will turn out to be correct, our data lend support to the thesis that preferences revealed by inexperienced subjects may be unreliable, as they are prone to change as experience with the game accumulates. This delivers two main messages. First, the literature on social preferences has somewhat downplayed the role of adaptive learning. Not only subjects participating in social dilemma experiments need to learn about other subjects’ choices: they also need to learn their own true preferences^33,34^. This has consequences that go well beyond the scope of the study of the decay of cooperation in anonymous PGG’s. Studies on social norms and social preferences^35,36^ show that a large fraction of the subjects believes reciprocally cooperative behavior to be the normatively appropriate behavior in a PGG. Our results suggest that these normative preferences should be taken with a pinch of salt, as subjects may change their mind as they gain experience with the game.

The second message is that subjects’ faulty understanding of the game in the early stages of the experiment not necessarily manifest itself as mere noise, hence it is particularly hard to detect. If all subjects who needed to learn how to play the game had initially chosen randomly, the increase of unconditional defectors would have been accompanied by a parallel decrease of individuals whose conditional contribution schedule follows a random pattern. However, this is not what we observe. Rather, we see that a substantial part of the change in the composition of the sample is due to subjects who are initially labeled as perfect reciprocators and eventually switch to unconditional defection. As long as these observations can be explained in terms of adaptive learning rather than by repeated game considerations, this implies that the subjects who are most likely to change their mind during the experiment are precisely the ones whose initial choices are easier to explain in terms of reciprocity, and hence would not be labeled as “confused”.

It is left to future research to study how adaptive and social learning combine to determine the evolution of subjects’ preferences, whether there are individual differences, and how the game used in the experiment may affect learning. An interesting issue is how, and if, one can isolate learning effects from repeated game considerations, as in both cases time and repetition play a crucial role. Most importantly, more research is needed to ascertain whether adaptive and social learning may eventually erode any form of pro-social behavior in experimental settings.

## Supplementary information

Supplementary Information.

## Data Availability

Our data, the programs used in the experiment, and the scripts to reproduce graphs and tables are publicly available on the GitHub repository of the study: https://www.github.com/seyhunsaral/stabilitycondcoop.
