# Supplementary material for: The stability of conditional cooperation: beliefs alone cannot explain the decline of cooperation in social dilemmas
Source: Sci Rep. 2020 Aug 12;10:13610. doi: 10.1038/s41598-020-70681-z (PMC7423935; doi:10.1038/s41598-020-70681-z)
Supplement: Supplementary file 1 — Supplementary Information. [file 41598_2020_70681_MOESM1_ESM.pdf]

Supplementary Information for

The Stability of Conditional Cooperation: Belifs Alone Cannot Explain the Decline of Cooperation in Social Dilemmas

Luciano Andreozzi * Matteo Ploner * Ali Seyhun Saral †‡

April 2020

*Department of Economics, University of Trento, 38122 Trento, Italy.
†Max Planck Institute for Research on Collective Goods, 53113, Bonn, Germany
‡To whom correspondence should be addressed. E-mail: saral@coll.mpg.de

Classification of Conditional Strategies

We denote each conditional strategy by three letters that represent the conditional responses to L, M, and H, respectively. Since there are only three responses, in total 27 different conditional strategies are possible. As the conditional strategies in our game are simple, the conditional type classification is rather self-evident; thus, we classify conditional types without the need of a calculation or a subjective evaluation.

We say that a player is selfish if he/she maximizes his/her own payoff (LLL); *conditional cooperator* if he/she increases its contribution monotonically. A special case of this type of strategy is the *perfect conditional cooperator* (LMH), who responds to the counterpart with the same action the counterpart played. We refer to conditional cooperators who are not perfect conditional cooperators as *imperfect conditional cooperators.*[1] We call a strategy *hump-shaped* if the most generous response of the player is a response to M (LML, LHL, LHM, MHL, MHM). We classified conditional strategies that do not fit any of the definitions above as *other* type. Figure 1 demonstrates the conditional strategies graphically and their classifications.

[1]Please note that we use the term "imperfect" in different sense than "selfish-biased. For instance the generous strategies such as MMH and MHH are "imperfect" since they do not copy the opponents' actions, although they are generous. For our data, this distinction do not make any difference, since no subject uses either of these imperfect conditional and generous strategies.

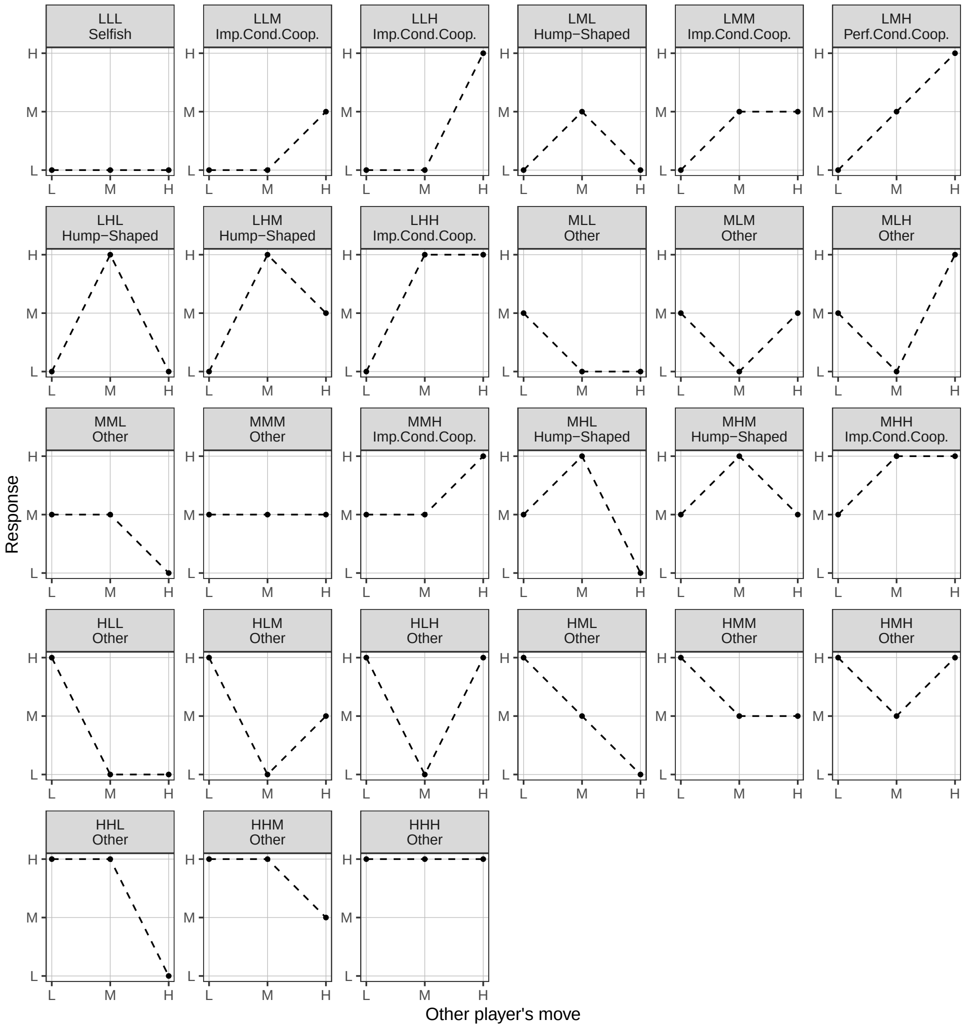

Figure 1: All possible conditional strategies and their classifications

Additional Figures

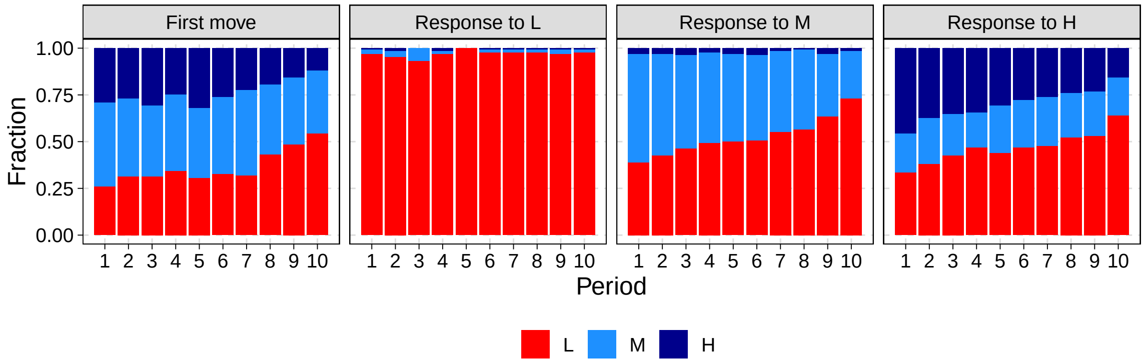

Figure 2: Average fraction of actions chosen as the first player and as the second player for as a conditional response

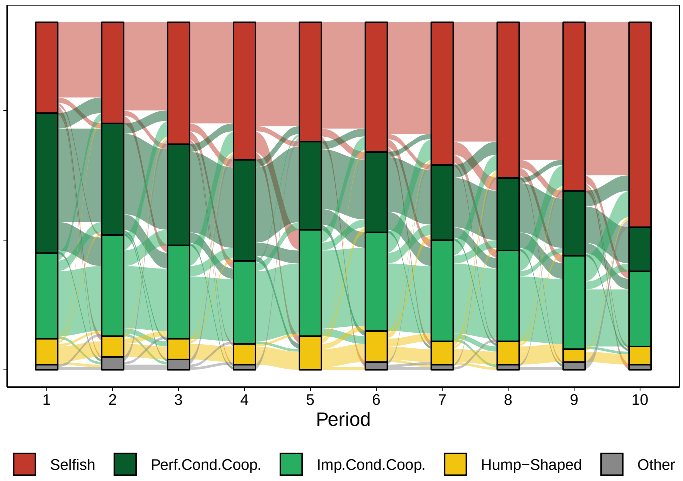

Figure 3: Fraction of conditional strategy types and transitions between types

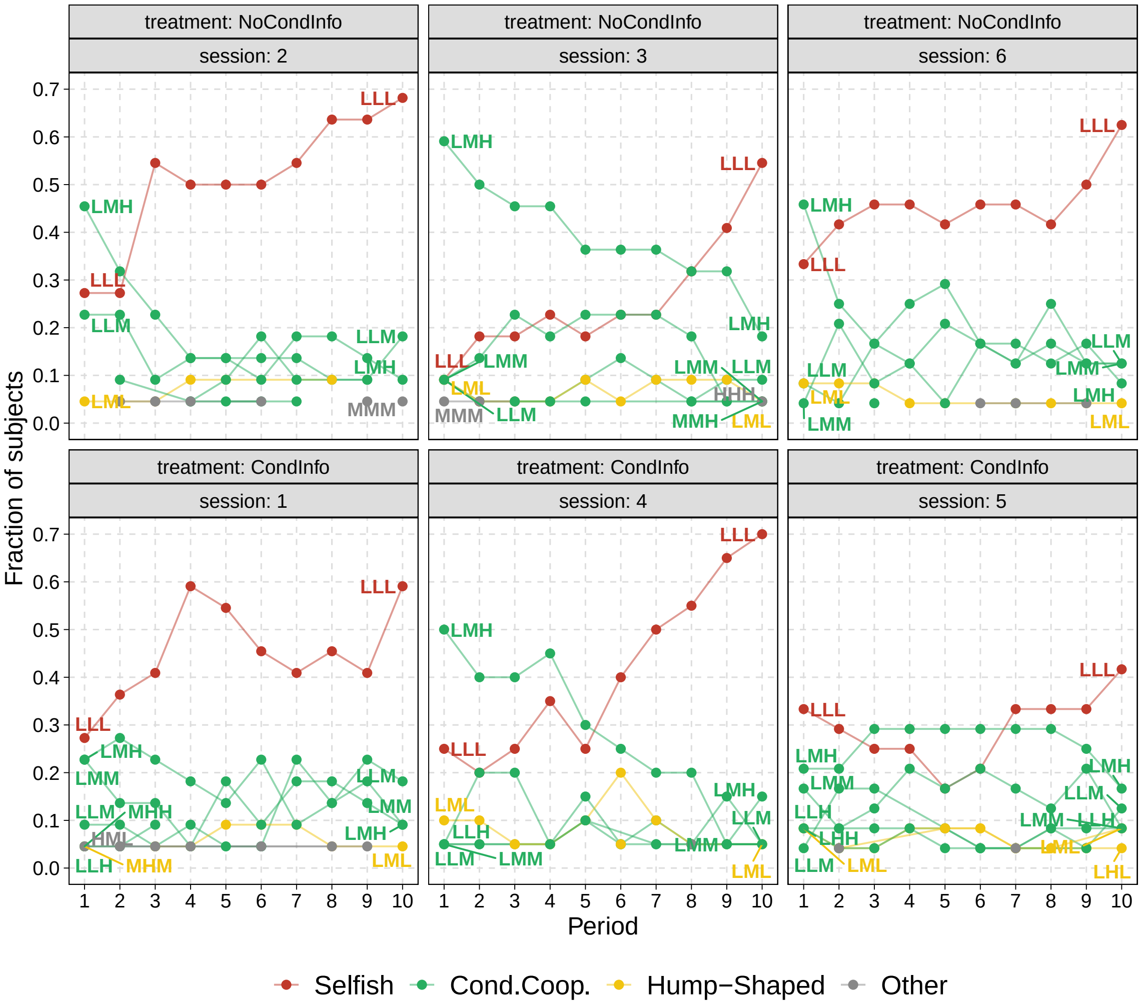

Figure 4: Fraction of conditional strategies by session and treatment

Belief Elicitation Method

We used the Quadratic Scoring Rule[1] to elicit subjects' beliefs on the opponents' action in an incentive-compatible manner. To increase the comprehension, we used an alternative presentation of the Quadratic Scoring Rule [2]. We designed an interface which contains a slider for each possible outcome to inform subjects about their possible earnings from the belief elicitation task. We also provided a table in the printed instructions which shows the relevant reward and penalty for the probability assigned

to an action.

The reward of a subject from one choice in the belief elicitation task in which a subject revealed his/her guess as $p = (p_L, p_M, p_H)$ and action $j \in \{L, M, H\}$ is chosen by the opponent, denoted $Q_A(p)$, and calculated as:

$$Q_j(p) = \alpha + \beta - \beta(1 - p_j)^2 - \beta \sum_{i \neq j} (p_i)^2. \quad (1)$$

We used parameters $\alpha = 20$ and $\beta = 20$ in our experiment, which give each subject the possibility to earn between 0 Experimental Currency (ECU) and 40 ECU in the belief elicitation task stage. For instance, if a subject assigns a 100% probability to the action chosen by the counterpart, he/she receives 40 ECU as a reward and 0 tokens as a penalty, which adds up to 40 ECU. Similarly, if a subject assigns a 50% probability to the action chosen by the counterpart and a 25% probability for the other two actions, he receives 35 ECU as reward and 5 ECU as a penalty for each of the other alternatives that are not selected, which adds up to 25 tokens received from the belief task for this particular action.

Table 1 shows the rewards and penalties table given to subjects.

Table 1: Rewards and Costs of Correct Beliefs according to Quadratic Scoring Rule

Assigned Probability(%)	*Reward if chosen (EC)*	*Cost if not chosen (EC)*
100	40	20
95	39.95	18.05
90	39.8	16.2
85	39.55	14.45
80	39.2	12.8
75	38.75	11.25
70	38.2	9.8
65	37.55	8.45
60	36.8	7.2
55	35.95	6.05
50	35	5
45	33.95	4.05
40	32.8	3.2
35	31.55	2.45
30	30.2	1.8
25	28.75	1.25
20	27.2	0.8
15	25.55	0.45
10	23.8	0.2
5	21.95	0.05
0	20	0

Notes on Beliefs

Figures 5 to 8 demonstrate the aggregated beliefs and the actual choices made by subjects. We concentrate on the two most interesting cases, in which beliefs concern the first player's unconditional choice (Figure 5) and the second player's choice conditional to the first player choosing the high transfer H (Figure 8). Figure 5 reveals that, on average, subjects' beliefs about the first player's choices are remarkably accurate in the initial rounds and show no tendency of becoming more or less accurate in later rounds. Figure 8 reveals that in the first stages of the game subjects are also successful at guessing the second player's transfer in response to an

high transfer. Interestingly, however, their guesses become *less* accurate as the game unfolds, as they become more selfish than they think the other subjects are.

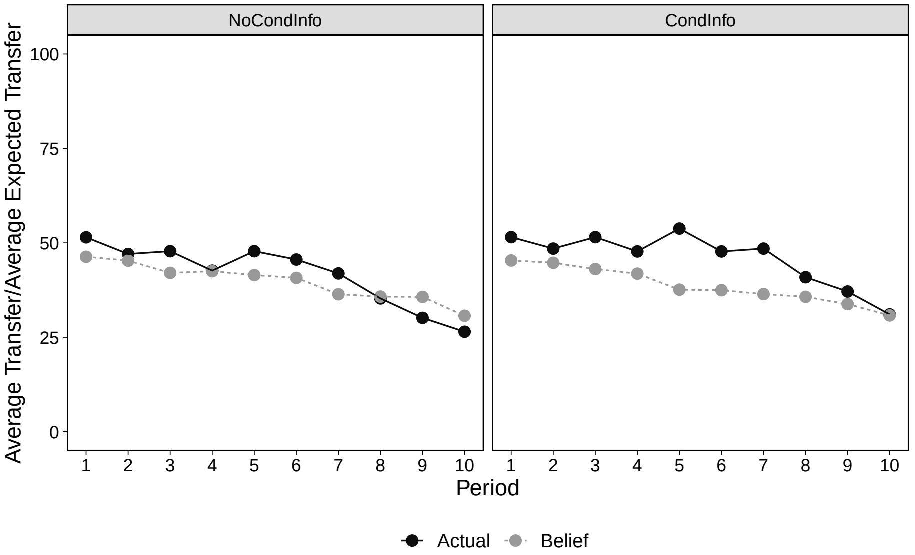

Figure 5: Mean Unconditional Transfer and Beliefs on the Expected Transfers in Each Treatment.

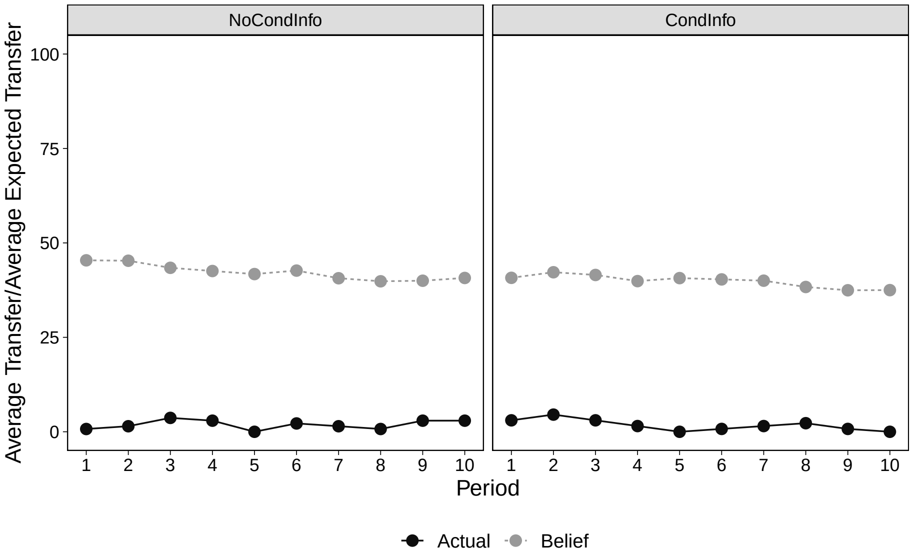

Figure 6: Mean Conditional Transfers and Beliefs on the Expected Transfers in Response to L in Each Treatment.

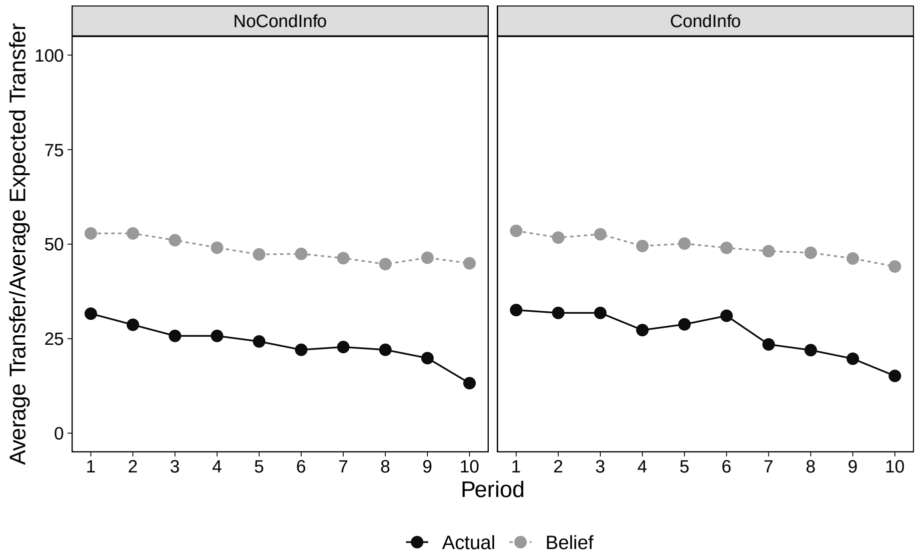

Figure 7: Mean Conditional Transfers and Beliefs on the Expected Transfers in Response to M in Each Treatment.

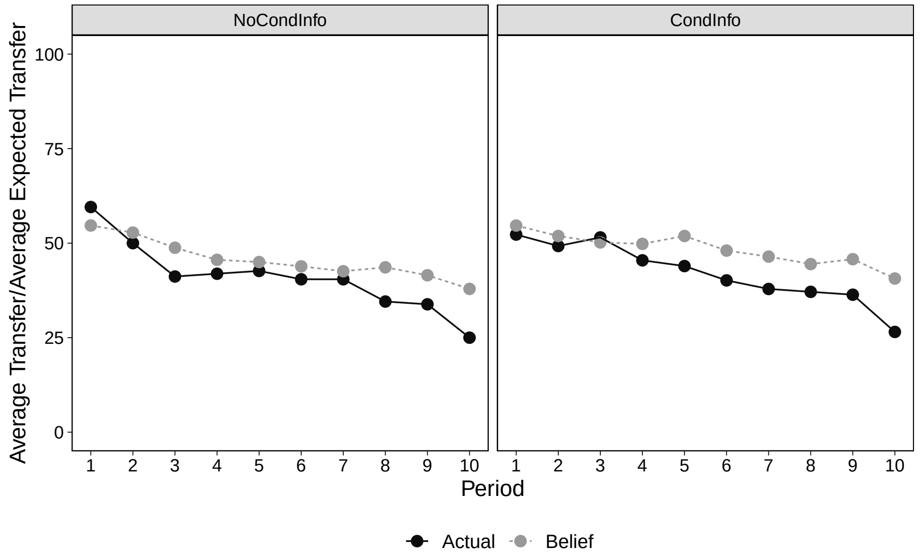

Figure 8: Mean Conditional Transfers and Beliefs on the Expected Transfers in Response to H in Each Treatment.

Comparison of the Proportion of Selfish and Conditional Types in Two Treatments

Period	*CondInfo*	*NoCondInfo*	p-value
1	0.62	0.68	0.62
2	0.61	0.62	1.00
3	0.62	0.50	0.22
4	0.53	0.53	1.00
5	0.56	0.56	1.00
6	0.47	0.56	0.39
7	0.48	0.53	0.73
8	0.47	0.47	1.00
9	0.50	0.41	0.39
10	0.36	0.32	0.76

Table 2: Proportions of Conditional Cooperators in Two Treatments and p-value of Chi-squared Test of Equal Proportions. We see no difference between treatments in any period.

Period	*CondInfo*	*NoCondInfo*	p-value
1	0.29	0.24	0.62
2	0.29	0.29	1.00
3	0.30	0.40	0.34
4	0.39	0.40	1.00
5	0.32	0.37	0.67
6	0.35	0.40	0.69
7	0.41	0.41	1.00
8	0.44	0.46	0.99
9	0.45	0.51	0.60
10	0.56	0.62	0.62

Table 3: Proportion of Selfish Players in Two Treatments and p-value of Chi-squared Test of Equal Proportions. We see no difference between treatments in any period.

Robustness Checks

Alternative Specifications

Random effects just at the individual level

	Model 1	Model 2
(Intercept)	$1.48\ (0.44)^{***}$	$1.34\ (0.47)^{**}$
period	$-0.24\ (0.03)^{***}$	$-0.22\ (0.04)^{***}$
treatment *NoCondInfo*	$-0.06\ (0.58)$	$0.22\ (0.66)$
period:treatment *NoCondInfo*		$-0.05\ (0.06)$
AIC	1223.76	1225.04
BIC	1244.56	1251.04
Log Likelihood	−607.88	−607.52
Num. obs.	1340	1340
Num. groups: subject	134	134
Var: subject (Intercept)	9.81	9.84

$^{***}p < 0.001$, $^{**}p < 0.01$, $^{*}p < 0.05$

Table 4: Alternative specification 1 - Mixed-Effects Logistic Regression: Dependent Variable: *isCondCoop*: The binary variable is equal to 1 when a subject is a conditional cooperator and equal to 0 otherwise.

Random effects just at the session level

	Model 1	Model 2
(Intercept)	$0.69\ (0.23)^{**}$	$0.64\ (0.25)^{*}$
period	$-0.11\ (0.02)^{***}$	$-0.10\ (0.03)^{***}$
treatment *NoCondInfo*	$-0.00\ (0.28)$	$0.11\ (0.36)$
period:treatment *NoCondInfo*		$-0.02\ (0.04)$
AIC	1810.66	1812.39
BIC	1831.46	1838.39
Log Likelihood	-901.33	-901.19
Num. obs.	1340	1340
Num. groups: session	6	6
Var: session (Intercept)	0.10	0.10

$^{***}p < 0.001$, $^{**}p < 0.01$, $^{*}p < 0.05$

Table 5: Alternative Specification 2- Mixed-Effects Logistic Regression: Dependent Variable: *isCondCoop*: The binary variable is equal to 1 when a subject is a conditional cooperator and equal to 0 otherwise.

Two-Way Clustered Random Effects

We implement here a multi-way clustering using the method suggested by Cameron, Gelbach, & Miller [3], which involves clustering on 2D+1 dimensional combinations,

	(1)	(2)
Constant	0.640**	0.691***
	(0.259)	(0.259)
period	−0.099**	−0.109**
	(0.049)	(0.049)
treatmentNoCondInfo	0.080	−0.021
	(0.425)	(0.425)
period:treatmentNoCondInfo	−0.018	
	(0.055)	
Note:	*p<0.1; **p<0.05; ***p<0.01	

Two-way Clustered Random Effects

We implement here cluster bootstrapping (also known as the block bootstrap) for variance/covariance matrices, following Cameron, Gelbach, & Miller [4].

	(1)	(2)
Intercept	0.640***	0.691***
	(0.147)	(0.104)
period	−0.099***	−0.109***
	(0.026)	(0.016)
treatment *NoCondInfo*	0.080	−0.021
	(0.239)	(0.161)
period:treatment *NoCondInfo*	−0.018	
	(0.033)	
Note:	*p<0.1; **p<0.05; ***p<0.01	

Two-step difference GMM (DPD)

To take into account the persistence of types, we estimate a two-step difference GMM with lagged type (past period) and period as explanatory variables (time invariant variables are omitted). The dependent variable is, like in Table 2 of the paper, a dichotomous variable capturing conditional type. The outcome of the regression estimates (obtainted by *xtabond2* command in Stata) are reported below.

Intercept	0.411***
	(0.142)
CC(L1)	0.204***
	(0.055)
period	−0.035***
	(0.005)
	AB AR(1):p< 0.001
	AB AR(2): p= 0.262
	H overid.: p= 0.263
Note:	*p<0.1; **p<0.05; ***p<0.01

As the regression outcomes show, past type positively predicts current type. However, the period variable has still a significant negative impact on the likelihood of being a conditional type.

Statistical Methods

The statistical analysis was run in the *R environment* [5] and our raw data were imported with the *zTree* package [6]. Figures in the Results section are produced via the *ggplot2* package [7]. In Table 2 we report p-values obtained of Pearson's Chi-squared tests with Yates' continuity correction on two-way frequency tables. Due to small sample size, we obtained p-values with Monte-Carlo simulation with 2000 replicates. None of the significance levels have changed according to simulated p-values. Thus we report non-simulated values. Table 3 summarizes the outcomes of a Generalized Linear Mixed Model (GLMM) fitted by maximum likelihood (Laplace Approximation) via the *lme4* package [8]. Given the dichotomous nature of the dependent variable, we adopted a binomial family specification with a logit link function. To account for repeated choices over rounds, we added a random effect at the individual level. The Akaike information criterion (AIC) is provided for the sake of model comparison.

Subject Screens (English Translation)

Figure 9-16 shows the translations of the subject screens into English. The original experiment is conducted in English and z-Tree [9] files are available with zBrac [10] compatible treatment and language files.

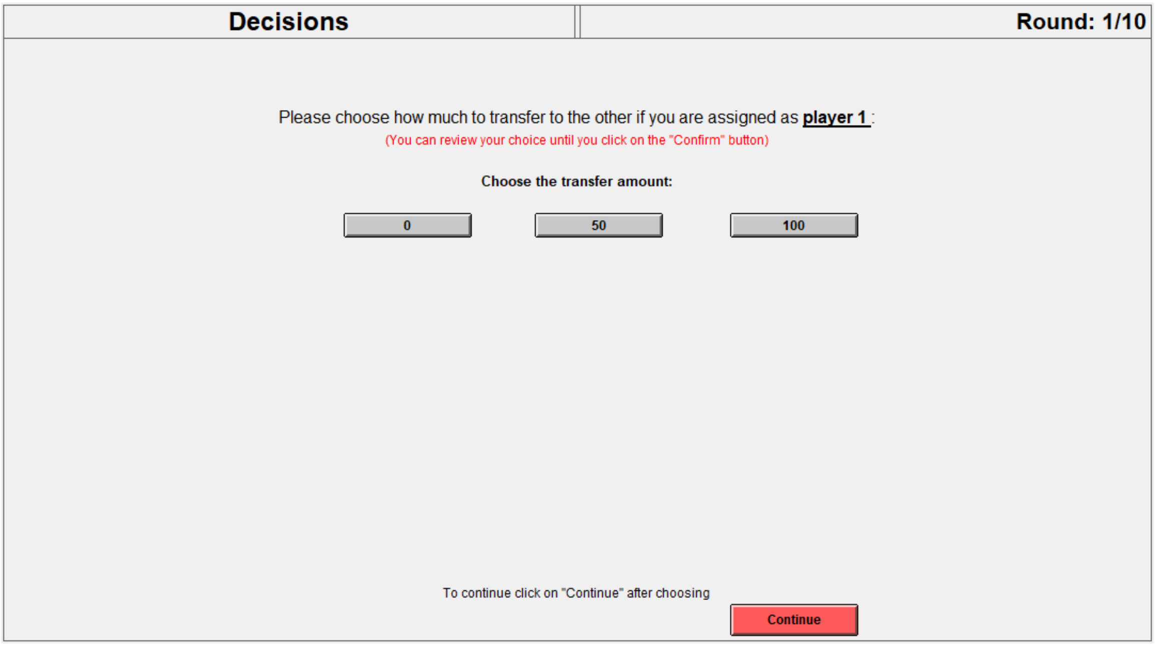

Figure 9: Decision Screen as the first player

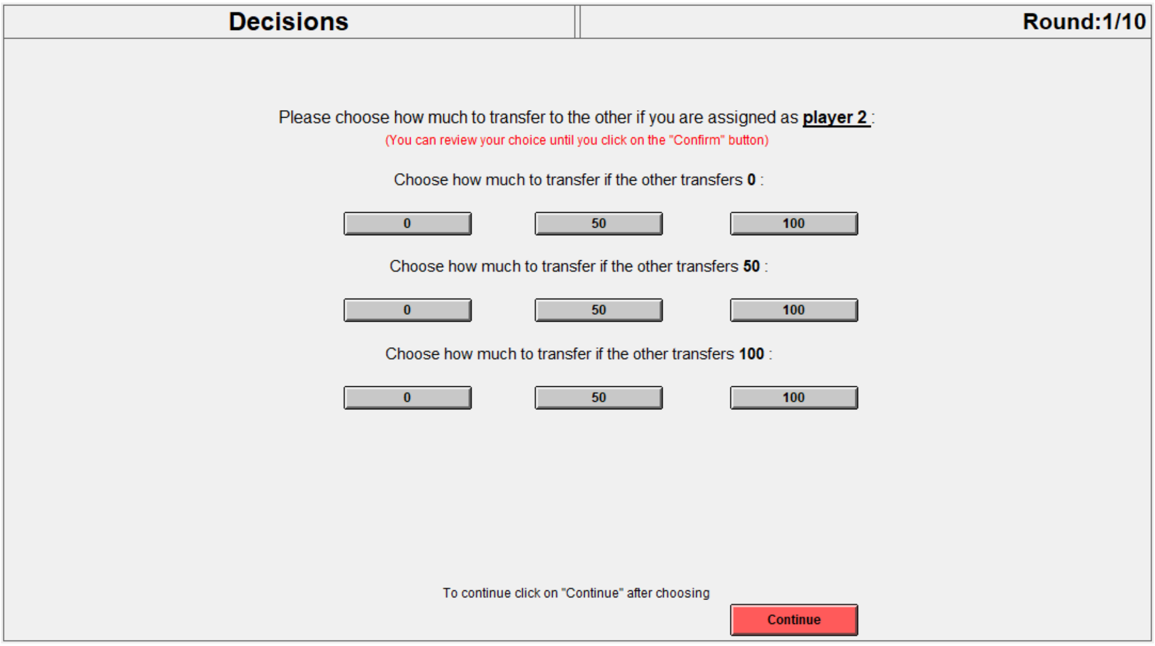

Figure 10: Decision Screen as the second player

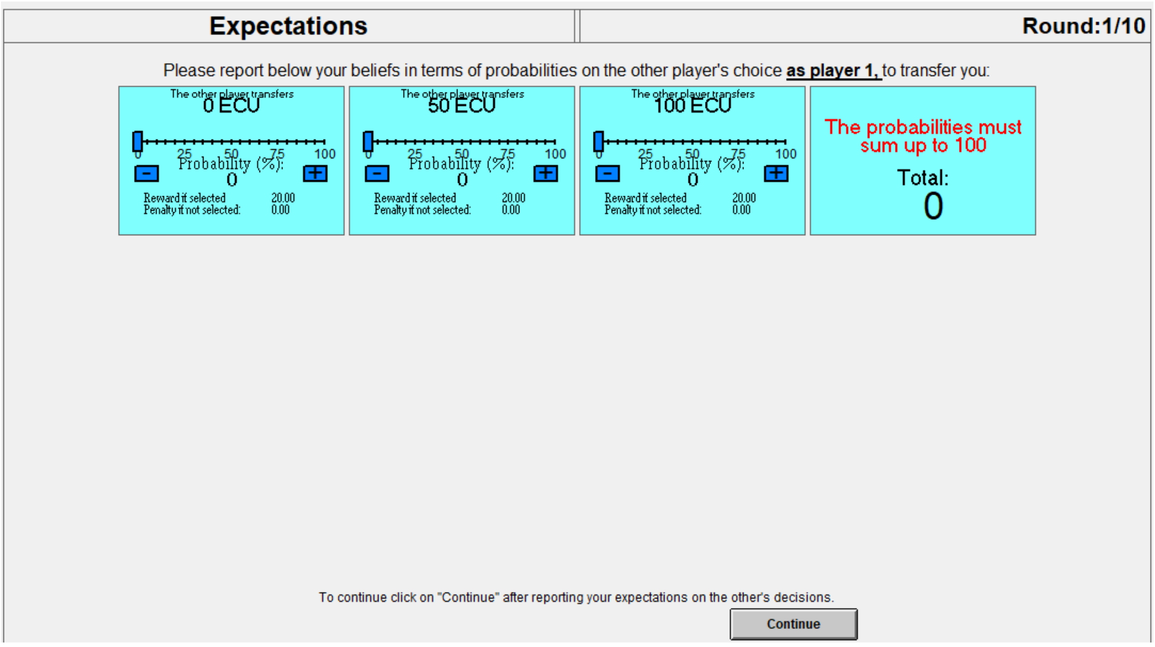

Figure 11: Belief elicitation: Beliefs on the opponent's actions as the first player

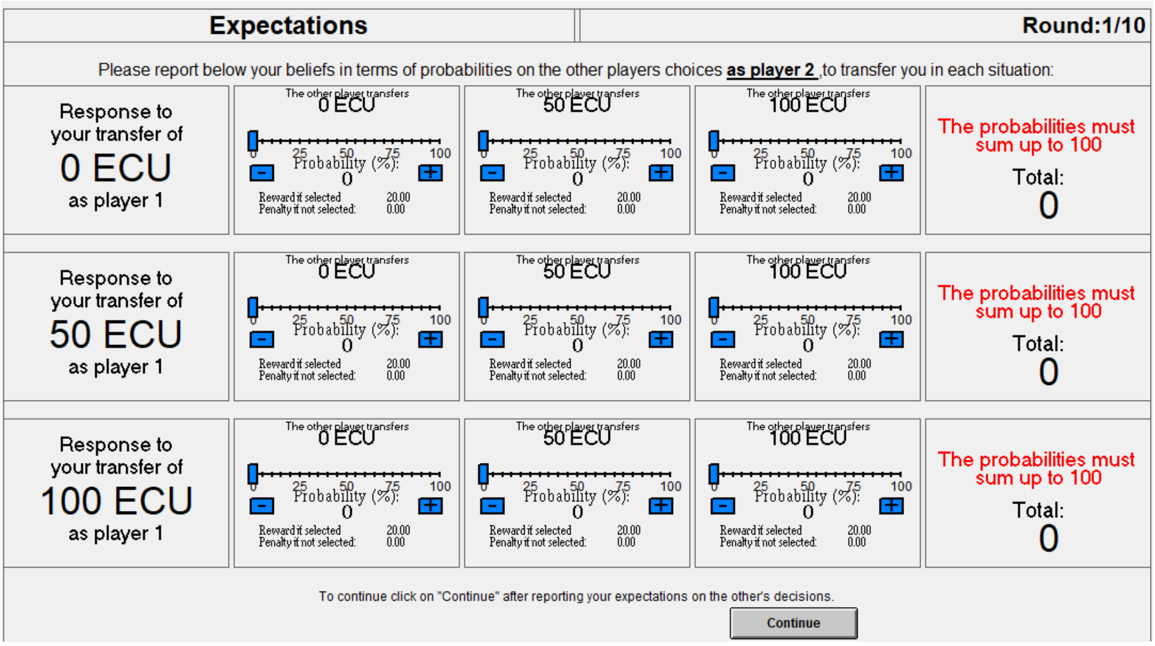

Figure 12: Belief elicitation: Beliefs on the opponent's actions as the second player

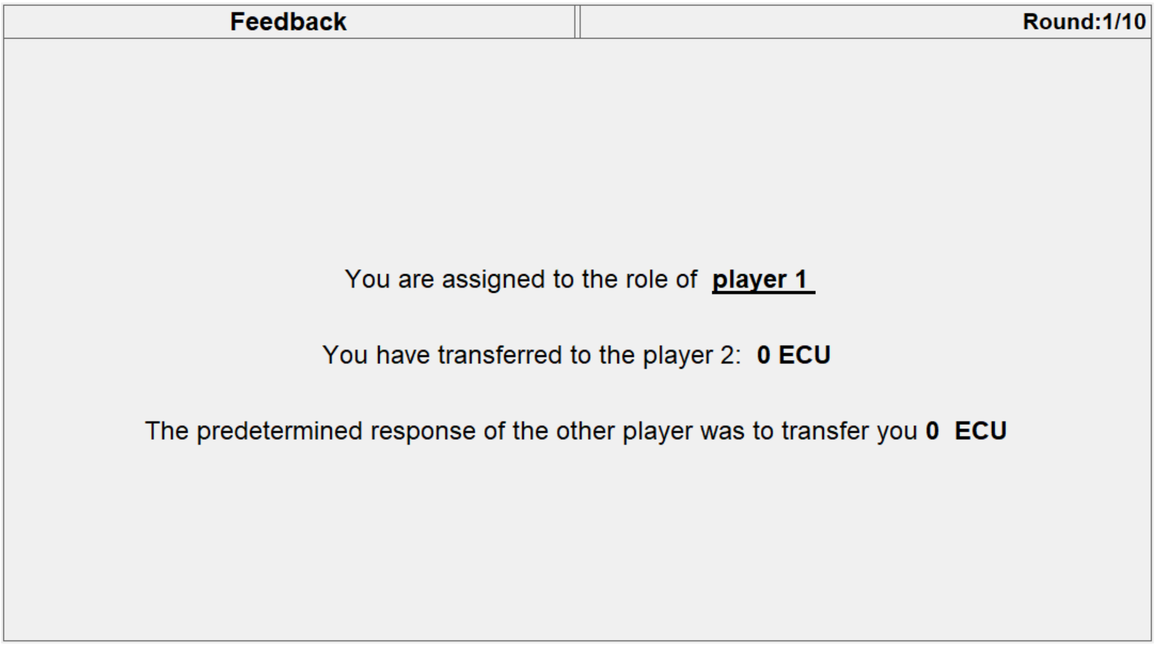

Figure 13: Feedback for the first player in *NoCondInfo* treatment

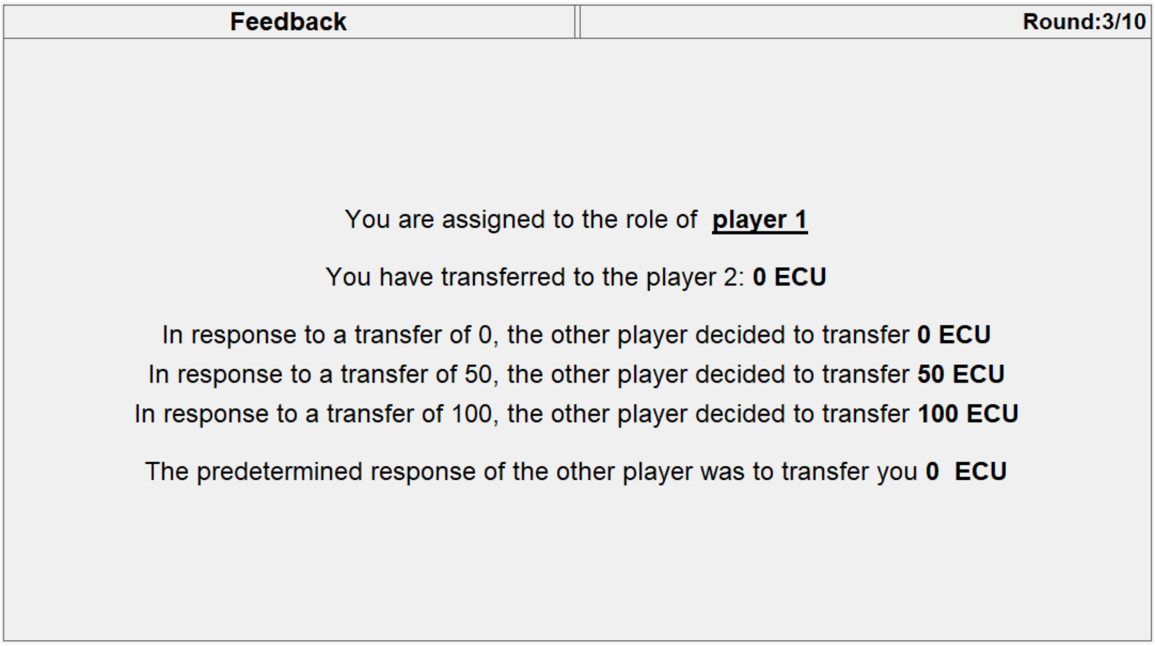

Figure 14: Feedback for the first player in *CondInfo* treatment

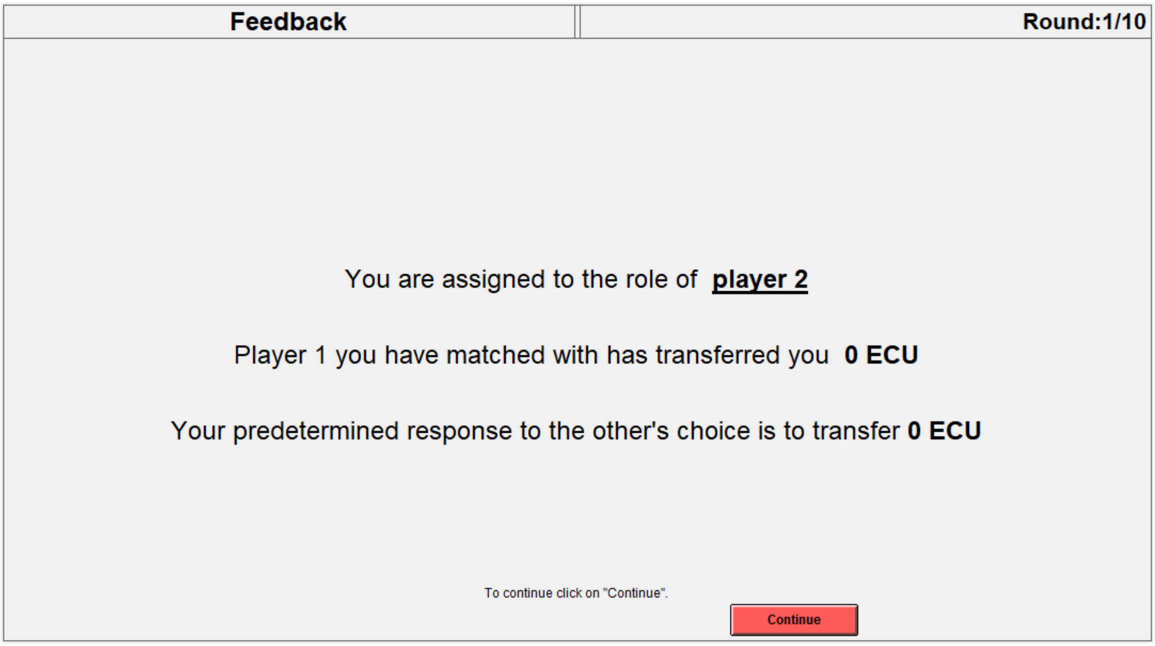

Figure 15: Feedback for the second player

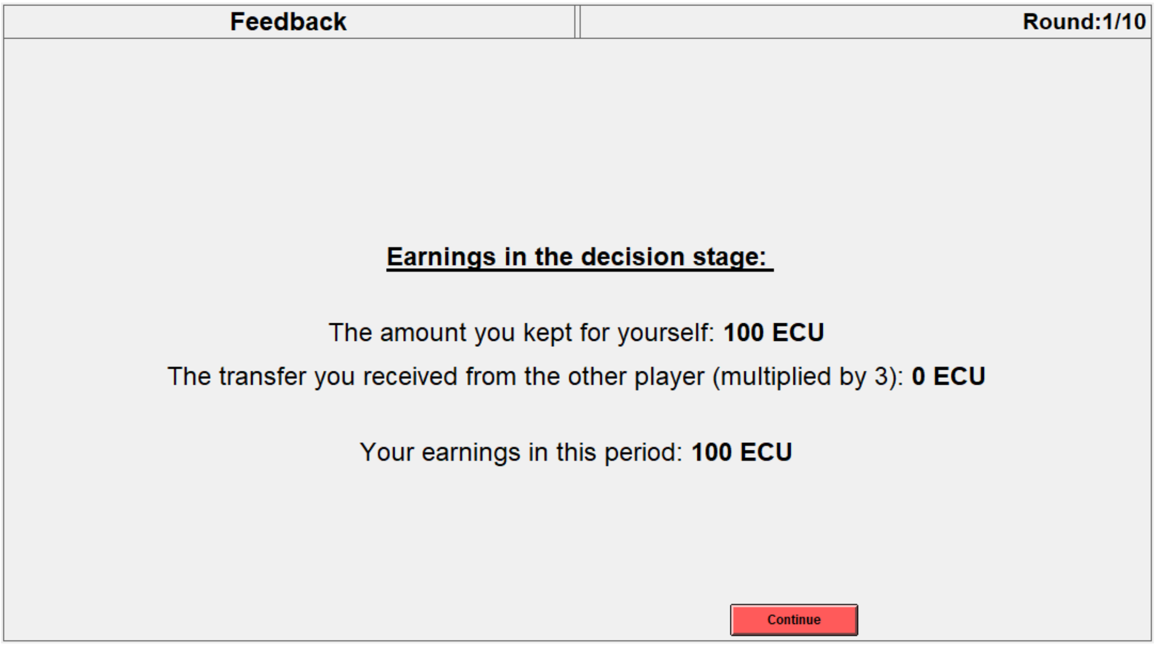

Figure 16: Payoff feedback

English translation of the written instructions

Instructions

General Information

Welcome to the experiment and thank you for your participation. This experiment is funded by **University of Trento** and all the data acquired will be used for scientific purposes.

From now on please remain silent, do not communicate with other participants and raise your hand if you have any question. The use of cell phones and of any other means of communication is forbidden. Please note that those violating these rules will be excluded from the experiment itself and all ensuing payments.

All data collected are held anonymous.

In this experiment, you can earn money depending on your decisions and those of other participants.

You will receive your payment in cash at the end of the experiment. You will be given **3 EUR** for taking part in the experiment. In addition, you may earn more according to the procedure explained below. During the experiment all the payments will be in **"Experimental Currency (ECU)"**. After the experiment, your earnings in ECU will be converted in Euros at the following conversion rate:

1 ECU = 0.05 EUROS

In some parts of the experiments, you will be interacting with other participants. During and after the experiment, your decisions and those of other participants will remain anonymous. This means that your identity of and that of the participant(s) you will interact with will be kept secret. The payment you will earn from the experiment will be given personally and will not be revealed to other participants. Moreover all the data acquired from the experiment will not be associated to the participants' identity.

Structure

The experiment is made of 10 repeated rounds identical to each other and each period will consist of two stages in which you will make decisions: **Decision Stage** and **Expectations Stage**. Prior to 10 identical periods of these two stages, there will be 4 rounds of a **Training Stage,** where you will become familiar with the experiment. At the end of the 10th round, you will go through a **Questionnaire Stage** where you will be asked to give feedback about the experiment and to answer a few questions.

The general structure of the experiment can be summarized by the table:

**Stage Name**	**Repetition**
Training Stage	**x4**
**Expectations Stage**	**x10**
**Decision Stage**	
Questionnaire	x1
Payments	x1

Procedure

Decision Stage

In this stage, you will be matched anonymously with a random participant in the lab. In each round, you will have an initial endowment of **100 ECU** and you will be asked to make transfer decisions to the matched participant, as described below.

In each pair, one of the players will be Subject **1** and the other will be Subject **2.** *However, you will make your choices both as Subject 1 and as Subject 2 in each round***.** After you and your partner have made your choices as Subject 1 and as Subject 2, the roles will be assigned and decisions will be implemented accordingly.

Subject 1 will have the option to transfer **0**, **50** or **100 ECU** to the other and to keep the rest for himself/herself. The amount that Subject 1 transfers will be multiply by **3.** This means that if Subject 1 selects to transfer **0**, he/she will keep all 100 ECU to himself/herself and the other will get nothing; if he/she selects to transfer **50 ECU** to the other, he/she will keep the **50 ECU** to himself/herself and the other will receive **150 ECU;** finally, if he/she decides to transfer **100 ECU,** he/she will keep nothing and the other will receive **300 ECU.** The decision screen of Subject 1 will be the following:

[First Player Decision Screenshot Here]

Also, **Subject 2** will have the option to transfer **0, 50** or **100 ECU,** as explained above. Similarly to Subject 1, the amount transferred by Subject 2 to Subject 1 will also be multiplied by **3.** However, Subject 2 will have the opportunity to choose his/her transfer to the other, conditional upon what Subject 1 chose transfer.

So Subject 2 will make three decisions:

To transfer **0, 50** or **100** ECU, if the other chooses **0 ECU;**
To transfer **0, 50** or **100** ECU, if the other chooses 5**0 ECU;**
To transfer **0, 50** or **100** ECU, if the other chooses **100 ECU;**

The actual transfer of Subject 2 will be the one made in correspondence to the actual choice of Subject 1.

The decision problem of Subject 2 will be shown in a decision screen like the following:

[Second Player Decision Screenshot Here]

As both subjects have 100 ECU and the amount transferred will be multiplied by 3, the earnings for both players will be as in the following table:

		**Choice of Subject 2 conditional upon choice of Subject 1**		
		**0 ECU**	**50 ECU**	**100 ECU**
**Choice of Subject 1**	**0 ECU**	Subject 1 earns 100 ECU,<br>(100 kept, 0 received)<br><br>Subject 2 earns 100 ECU.<br>(100 kept, 0 received)	Subject 1 earns 250 ECU,<br>(100 kept,150 received)<br><br>Subject 2 earns 50 ECU.<br>(50 kept, 0 received)	Subject 1 earns 400 ECU,<br>(100 kept,300 received)<br><br>Subject 2 earns 0 ECU.<br>(0 kept, 0 received)
	**50 ECU**	Subject 1 earns 50 ECU,<br>(50 kept, 0 received)<br><br>Subject 2 earns 250 ECU.<br>(100 kept, 150 received)	Subject 1 earns 200 ECU,<br>(50 kept, 150 received)<br><br>Subject 2 earns 200 ECU.<br>(50 kept, 150 received)	Subject 1 earns 350 ECU,<br>(50 kept, 300 received)<br><br>Subject 2 earns 150 ECU.<br>(0 kept, 150 received)
	**100 ECU**	Subject 1 earns 0 ECU,<br>(0 kept, 0 received)<br><br>Subject 2 earns 400 ECU.<br>(100 kept, 300 received)	Subject 1 earns 150 ECU,<br>(0 kept, 150 received)<br><br>Subject 2 earns 350 ECU.<br>(50 kept, 300 received)	Subject 1 earns 300 ECU,<br>(0 kept, 300 received)<br><br>Subject 2 earns 300 ECU.<br>(0 kept, 300 received)

After both subjects have made their choices both as Subject 1 and as Subject 2, the computer will randomly assign roles, and the choice made by Subject 1 and the choice made by Subject 2 conditional upon that of Subject 1 will be implemented. The matching of choices will define the earnings in the round.

Note that in each period you have the same chance of being Subject 1 or 2. Therefore it is likely that in some rounds you will be selected as Subject 1 and in others as Subject 2.

The interaction will be repeated 10 times and in each round you will be randomly matched with another participant in the session.

At the end of the experiment, one of the rounds will be selected and the earnings from that round will define your final payment.

Feedback about the Decisions

[TREATMENT NoCondInfo]: After each round, you will receive feedback about the other's decisions: if you are selected to be Subject 1, you will see the other's response to your choice, and Subject 2 will see your choice. If you are selected to be Subject 2, you will see the other's choice and Subject 1 will see your corresponding response to his/her choice. Feedback will be delivered in a screenshot like the following:

[Treatment NoCondInfo Feedback Screenshot Here]

[TREATMENT CondInfo]: After each round, you will receive a feedback about the other's decisions: if you are selected to be Subject 1, you will see the other's conditional choice to each possible choice of yours, and Subject 2 will see your choice. If you are selected to be Subject 2, you will see the other's choice and Subject 1 will see your conditional choices to each possible choice of him/her. Feedback will be delivered in a screenshot like the following:

[Treatment CondInfo Feedback Screenshot Here]

Summary of the Decision Stage

- Both subjects select the amount to transfer as Subject 1 (0 , 50 or 100 ECU).
- Both subjects select their choices as Subject 2 (0, 50, or 100 ECU conditional upon each 3 possible transfers of Subject 1).
- One subject will be selected as Subject 1, one subject will be selected as Subject 2, randomly by the computer.
- The choices of the two subjects are implemented.
- Subjects receive a feedback about the choices of the other.

Expectations Stage

In each round, before the Decision Stage, you will take part in the Expectations Stage. You will be asked to assess for each of the options available how likely it is that it is going to be chosen by the other. Since in each round the other has three options (to transfer 0 ECU, 50 ECU, or 100 ECU), you will be asked to report a probability for each option that the other has. The sum of probabilities must be equal to 100. The earnings in this stage are proportional to the accuracy of your estimate. You can report the probabilities either by using the sliders (dragging the blue handle to the left and right) or the plus and minus signs in each field.

Since in the Decision Stage each player will make four decisions, you will be asked to state your beliefs for each of the four different choices. One for the other's choice as Subject 1 (first screen) and three for the other's choice as Subject 2 (second screen).

You will see a screen like the one below to state your beliefs about the other's decision as Subject 1:

[First Player Expectations Screenshot Here]

After completing this screen, you will be shown a screen like the following to predict the other's choices as Subject 2:

[Second Player Expectations Screenshot Here]

For each decision taken by the other, you will earn an amount of money for the probability you assigned to the choice taken. The larger the probability you give to the chosen option, the more ECU you will earn as a reward (values are shown in the second column of the table below). However, for the probabilities you assign to a decision that is not chosen, you will incur a cost. The larger the probability you give to the unchosen option, the more ECU will be subtracted from your earnings (values are shown on the third column of the table below)

Since there are three options for each choice (to transfer 0, 50, or 100 ECU), you will get a reward for the right choice, while you will pay a cost for the other two choices, which will be subtracted from your earnings. The procedure to compute rewards and costs is the same for all choices and for all rounds.
The table below shows rewards and costs associated with each assigned probability:

**Assigned Probability to an option (%)**	**Reward if this option is chosen (ECU)**	**Cost if this option is not chosen (ECU)**
100	40	20
95	39.95	18.05
90	39.8	16.2
85	39.55	14.45
80	39.2	12.8
75	38.75	11.25
70	38.2	9.8
65	37.55	8.45
60	36.8	7.2
55	35.95	6.05
50	35	5
45	33.95	4.05
40	32.8	3.2
35	31.55	2.45
30	30.2	1.8
25	28.75	1.25
20	27.2	0.8
15	25.55	0.45
10	23.8	0.2
5	21.95	0.05
0	20	0

On the screens where you make your choice, you will be able to see the rewards and the costs for the probability you assign to each option and the total earnings as well. However, if you prefer, you can refer to the table during the experiment.

EXAMPLE:
Assume that you stated that the probability that the other chooses to transfer 50 ECU is 80%, the probability to transfer 0 ECU is 10%, and the probability to transfer 100 ECU is 10%. If the other actually chooses to transfer 50 for this decision:

You will receive the reward for the 80% assigned to the right choice, that is, 39.2 ECU.
You will pay the costs for the 10% assigned to the two wrong choices, that is, 0.2 ECU for each.
Thus, your earning will be 39.2 – 0.2 – 0.2 = 38.8 ECU.

EXAMPLE:
Assume that you stated that the probability that the other chooses to transfer 0 ECU is 50%, the probability to transfer 50 ECU is 30%, and the probability to transfer 100 ECU is 20%. If the other actually chooses to transfer 50 ECU for this decision:

You will receive the reward for the 30% assigned to the right choice, that is, 30.2 ECU.
You will pay the costs for the 50% assigned to the first wrong choice, that is, 5 ECU.
You will pay the costs for the 20% assigned to the second wrong choice, that is, 0.8 ECU.
Thus, your earning will be 30.2 – 5 – 0.8 = 24.4 ECU.

It can be seen that the maximum amount you can earn for your choice is 40 ECU when you assign 100% probability to an option chosen (40 – 0 – 0), and minimum amount you can eceive is 0 ECU by assigning 100% to an option that is not chosen (20 – 20 – 0).

Please note that with this mechanism it is in your interest to report your true beliefs.

At the end of the experiment, from all the guesses you make, one of the guesses will be selected and you will be paid according to the accuracy that guess. There will be no feedback after the expectations stages, and your earnings will be noted at the end of the experiment and will be added to your payment.

Summary of the Expectations Stage

- Both players state their beliefs about what the other will choose as Subject 1 and as Subject 2.
- In each round, players are rewarded for the probability given to the choice made by the other and will incur a cost for the probability given to the choice not made by the other.
- There will be no feedback after the Expectations Stage.

Training Stage

In order to get familiar with the Decision Stage and the Expectations Stage, there will be a training stage of 4 rounds. In this stage, instead being matched with participants in the lab, you will interact with a computer choosing randomly.

In the first two rounds, you will only go through the Decision Stage and in the other two rounds you will see the Expectations Stage first and the Decision Stage afterwards.

The choices made in the Training Stage do not contribute to your final earnings.

After the 4 rounds of the Training Stage, you will start the actual experiment interacting with other players for 10 rounds.

We have reached the end of the instructions. Please raise your hand if you have any question.

References

1. Brier, G. W. Verification of forecasts expressed in terms of probability. *Monthey Weather Review* **78,** 1–3 (1950).
2. Artinger, F., Exadaktylos, F., Koppel, H. & Sääksvuori, L. *Applying quadratic scoring rule transparently in multiple choice settings: A note* Jena economic research papers, 2010-2021. 2010.
3. Cameron, A. C., Gelbach, J. B. & Miller, D. L. Robust inference with multiway clustering. *Journal of Business & Economic Statistics* **29,** 238–249 (2011).
4. Cameron, A. C., Gelbach, J. B. & Miller, D. L. Bootstrap-based improvements for inference with clustered errors. *The Review of Economics and Statistics* **90,** 414–427 (2008).
5. R Core Team. *R: A Language and environment for statistical computing* R Foundation for Statistical Computing (Vienna, Austria, 2019).
6. Kirchkamp, O. Importing z-Tree data into R. *Journal of Behavioral and Experimental Finance* **22,** 1–2 (2019).
7. Wickham, H. *ggplot2: Elegant graphics for data analysis* (Springer, 2016).
8. Bates, D., Mächler, M., Bolker, B. & Walker, S. Fitting linear Mixed-effects models using lme4. *Journal of Statistical Software* **67,** 1–48 (2015).
9. Fischbacher, U. z-Tree: Zurich toolbox for ready-made economic experiments. *Experimental economics* **10,** 171–178 (2007).
10. Saral, A. S. & Schröter, A. M. zBrac - A multilanguage tool for z-Tree. *Journal of Behavioral and Experimental Finance* **23,** 59–63 (2019).
